# “Gongs Mobilization “Approach for Frozen Shoulder

**DOI:** 10.7759/cureus.30890

**Published:** 2022-10-31

**Authors:** Gauri Kariya, Pooja Dhage, Nikita S Deshmukh

**Affiliations:** 1 Musculoskeletal Physiotherapy, Ravi Nair Physiotherapy College, Datta Meghe Institute of Medical Science, Wardha, IND; 2 Musculoskeletal Physiotherapy, Ravi Nair Physiotherapy College, Datta Meghe Institute of Medical Sciences, Wardha, IND

**Keywords:** range of motion, discomfort, physical therapy, frozen shoulder, gongs mobilization

## Abstract

Discomfort and stiffness in the shoulder joint are the main causes of a frozen shoulder. The main contributing factor to frozen shoulder is typically co-morbid disorders like diabetes mellitus and hypertension. Adhesive capsulitis is another name for a frozen shoulder. Range of motion is the main aspect that is targeted when the illness slowly worsens. The three stages of a frozen shoulder are the freezing stage, the frozen stage, and the thawing stage. Physical therapy plays an important role in providing relief for this condition, but the usual conservative management is more time-consuming, so a patient with a frozen shoulder is managed along with Gong's mobilization and the usual conservative management is given for two weeks. This case report aims to show the result of Gong's mobilization in two weeks. Further, in this case report, the proper procedure for the Gong's mobilization is explained.

## Introduction

The frozen shoulder, which is also known as adhesive capsulitis, has a target on the shoulder joint. It influences 2-5% of the general population and causes a gradual loss of shoulder mobility [1]. The succession of processes from capsular inflammation and fibrosis to spontaneous clearance of this fibrosis is reflected in the many stages that FS goes through [2]. A frequent disabling condition known as "frozen shoulder" is characterized by shoulder discomfort and a gradual loss of shoulder motion. The prolonged clinical course of a frozen shoulder, which usually develops in conjunction with other systemic disorders or after periods of immobility, can be irritating for both patients and medical practitioners. Inflammation, new angiogenesis, and new nerve growth are all associated with this process, which leads to shoulder capsular fibrotic contractures and related clinical stiffness. The stage of the illness or the presence of concurrent shoulder pathology might affect how difficult it is to make a diagnosis, which is mainly reliant on physical examination. The efficiency of management techniques including physical therapy, therapeutic methods like steroid injections, anti-inflammatory drugs, hydrodilatation, and surgical procedures is yet unknown. Facilitating translational science should contribute to the creation of innovative treatments to enhance outcomes for those who suffer from this crippling ailment [3]. Frozen shoulder, also known as idiopathic shoulder stiffness, is a common condition of the glenohumeral joint that is characterized by an abrupt onset of pain and a gradual loss of range of motion. The fundamental origin of FS is still unclear, even though the condition’s histological alterations coincide with synovial inflammation and increasing capsular fibrosis. Scientists are divided about which treatment approaches are most likely to contribute to symptom improvement in this case, regardless of the disease's stage. This is because the illness frequently improves and the symptoms go away after a while. This case report focuses on explaining the scientifically researched conservative therapeutic choices for FS and, as a result, their chronological classification into the disease's distinctive three stages [4].

Gong’s mobilization

The method used to lessen discomfort and extend the joint range of motion is called joint mobilization [5,6]. The vast majority of joint issues are treated with it. Based on Maitland grades, mobilization procedures are carried out, and pressures are used based on the degree of discomfort. To enhance ROM by stretching soft tissues, several techniques, including distraction, compression, rolling, and spinning, are used [6]. With the shoulder in a dynamic posture, Gong's mobilization is given by antero-posterior glide before distracting the patient and having them do the limited movement. The study's major goal was to find out how Gong's mobilization helped patients with frozen shoulders in terms of discomfort, range of motion, and handicap [7]. For two weeks, Gong's mobilization was a mobilization intervention program [8]. As Gong's mobilization was used as an approach in this condition, the assessment was taken before using this approach as well as after using it and given in the tabular format afterward in this case report. A pilot study showed that Gong's mobilization can help people with frozen shoulders by reducing their discomfort, range of motion, and impairment [9]. Gong discovered that Gong's mobilization approach preserves the shoulder in a neutral posture at the end of its range of motion and is more effective in increasing shoulder internal rotation than anterior to posterior gliding [10].

Procedure for gongs mobilization

This case report's Gong's mobilization strategy was developed using guidelines provided by Gong et al. [11]. The troublesome shoulder joint was turned upward as the patient was positioned in a side-lying posture. The patient had a 90-degree abduction of the shoulder to keep the humerus upright, and the elbow was 90 degrees flexed. Now the therapist held one hand of the patient to apply pressure to the humeral head from anterior to posterior while maintaining the position of the patient's elbow at 90 degrees. The therapist then raised their own body while softly pushing on the shoulder joint's articular capsule. To retain the humerus' vertical axis, they achieved this while keeping shoulder abduction and elbow flexion at 90 degrees. The procedure was finished in about two to three minutes, and the articular capsule was gently squeezed for 10-15 seconds, then relaxed for five seconds. After slightly stretching the capsule, the therapist applied gentle pressure with one hand on the shoulder joint from anterior to posterior. This stopped the humerus and the somewhat enlarged articular capsule from being pulled vertically. While completing shoulder medial rotation with the other hand, the therapist stabilized the elbow. Maitland grades 3 and 4 then performed oscillation to increase the range of motion, and the grade 4 approach extended stretching for seven seconds.

## Case presentation

Patient observation

A 59-year-old female homemaker by occupation came to the physiotherapy department with complaints of pain in the shoulder while performing activities such as bathing, dressing, and combing. She had difficulty performing overhead activities of daily living and had painful movements. She is a known case of diabetes mellitus and hypertension. She visited an orthopaedician, and a physical examination was conducted, and an X-ray was also performed. After being conservatively managed, this patient felt relieved, but then she began to experience the same complaints, so she returned to the same orthopaedician, who referred her to physiotherapy OPD. After coming to physiotherapy, the OPD patient was diagnosed with stage-2 frozen shoulder.

Clinical findings

**Figure 1:**
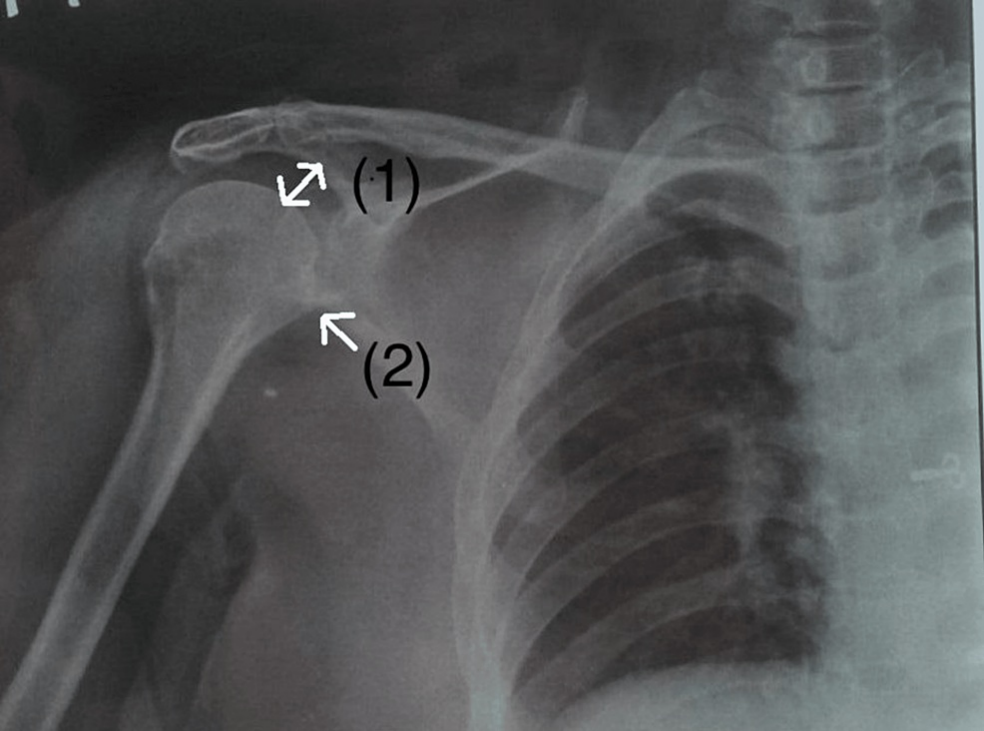
X-ray of the patient (i) Slight degenerative changes and (ii) slight decrease in the joint space which causes hypomobility.

The patient gave her consent. The diagnosis, physical examination, and course of treatment were all described to the patient. The patient was compliant, aware, and attuned to time, place, and person during the general examination. The patient’s vitals were taken and were hemodynamically stable. There was grade 1 tenderness present over the right side of the neck, and it felt tight, and the shoulder area was tense. Figure 1 is an X-ray of the patient.

Table 1 shows the Numerical Pain Rating scale (NPRS) for pain assessment before the intervention. Table 2 shows the NPRS scale for pain assessment after the intervention. Table 3 shows the ROM assessment before the intervention. Table 4 shows MMT scores before the intervention. Table 5 shows the MMT score after the intervention. Table 6 shows the ROM assessment after the intervention. Shoulder Pain and Disability Index (SPADI) is used to assess the outcome measures.​​ Total disability score of the patient is 38/80 and the total SPADI score is 68/130.

**Table 1:** Numerical Pain Rating scale for pain assessment before intervention

Sr.no	Score on rest	Score on activity
1	4/10	7/10

**Table 2:** Numerical Pain Rating Scale for pain assessment after intervention

Sr.no	Score on rest	Score on activity
1	2/10	4/10

**Table 3:** Range of motion assessment before intervention

Sr. No	Degrees of motion	Ranges in degrees
1	Shoulder flexion	0–102
2	Shoulder extension	0–38
3	Shoulder Internal rotation	0–37
4	Shoulder external rotation	0–70
5	Elbow flexion	0–115
6	Elbow extension	115–0

**Table 4:** Manual Muscle Testing scores before intervention

Sr.no	Muscle groups	Score
1	Shoulder flexors	3/5
2	Shoulder extensors	3/5
3	Elbow flexors	4/5
4	Elbow extensors	3/5

**Table 5:** Manual Muscle Testing score after intervention

Sr. no	Muscle groups	Score
1	Shoulder flexors	3+/5
2	Shoulder extensors	3+/5
3	Elbow flexors	4/5
4	Elbow extensors	4/5

**Table 6:** Range of motion assessment after intervention

Sr. no	Degrees of motion	Ranges in degrees
1	Shoulder flexion	0–175
2	Shoulder extension	0–55
3	Shoulder Internal rotation	0–60
4	Shoulder external rotation	0–80
5	Elbow flexion	0–130
6	Elbow extension	130–0

Therapeutic interventions

Goals of Intervention

**Figure 2:**
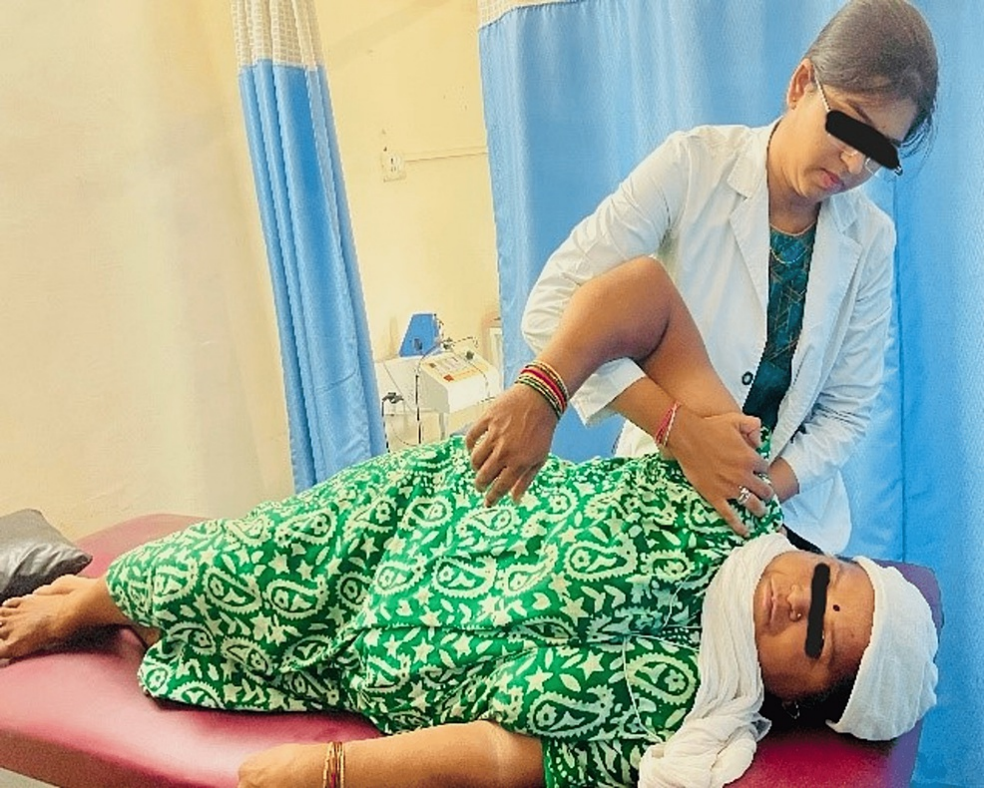
Therapist giving gongs mobilization

Patient education about the condition and how physiotherapy interventions are necessary to relieve the symptoms. Decrease score of pain on NPRS during rest as well as on activity. Improve and maintain range of motion (ROM) for all the affected motions. Improve and maintain strength in the patient in all the muscle groups of upper limb. Promote functional independence in the patient, make patient functionally independent. Initially 1/2 kg weight was used in strengthening then increase in gradual weight by 1/2 kg after every 4 days. Gong’s mobilization 10-15 repetitions [12] The intervention lasted for two weeks and consisted of five therapy sessions each week [13]. Scapular retraction exercises (Wall push ups) Posterior capsular stretch. Isometric shoulder external rotation for maintenance of muscle strength. Inferior glide to increase shoulder abduction. Following figure 2 shows therapist giving gongs mobilization

## Discussion

In all three stages of a frozen shoulder, there is an increase in discomfort and stiffness. Numerous physiotherapy techniques are used to treat frozen shoulders; however, this case study highlights the benefits of gong mobilization in addition to other conservative measures. Joint mobilization effectively treats reversible painful joints with restricted mobility and functionally fixed joints due to its neurophysiologic and mechanical effects: rhythmic oscillatory movements that stimulate type-2 dynamic mechanoreceptors and inhibit type-4 nociceptive receptors, as well as an effect on circulatory perfusion [8]. Additionally, improved outcomes are observed as a result of the mobilization that is administered near the conclusion of the range of motion, which keeps the shoulder joint in its natural posture throughout anterior to posterior gliding and yields an instant response. Therefore, Gong's mobilization technique may be used to increase shoulder joint ROM or reduce stiffness in the GH joint. When someone is supine, a common joint mobilization technique used to enhance the shoulder medial rotation range of motion is called anterior-to-posterior gliding. Gong's mobilization increases the range of motion for shoulder medial rotation by allowing shoulder medial rotation with the humeral head in a normal position against the glenoid cavity of the scapula. However, unlike when the body is in a static position, anterior-to-posterior gliding during dynamic movement does not maintain the humeral head in a normal posture [14] and is more effective in enhancing shoulder internal rotation than anterior-to-posterior gliding [12].

## Conclusions

A frozen shoulder is nowadays a commonly encountered condition that is seen in individuals of different age groups. Pain may worsen with each increasing stage or may remain persistent and may even radiate. This pain and discomfort affect the daily living activities of the patients, and they encounter various problems even in the simple day-to-day activities that they used to perform earlier. Many investigations, which include X-ray, MRI, or ultrasound, are of great importance in taking the therapist towards the diagnosis. To know the effectiveness of Gong's mobilization in this frozen shoulder case before and after intervention, the range of motion, MMT of the patient, and pain assessment before and after intervention with the help of NPRS were studied, which helped us to know the efficiency of this mobilization. Many studies suggest the use of various mobilization techniques and various other treatment protocols that stop the progression of the disease and provide relief from symptoms. The goal of this case report is to make readers aware of this type of mobilization technique and recognize this method as a new and recent advance for the frozen shoulder. It also aims to provide sufficient treatment to provide relief from the above condition and increase mobility for the above condition.

## References

[1] Lho YM, Ha E, Cho CH (2013). Inflammatory cytokines are overexpressed in the subacromial bursa of frozen shoulder. J Shoulder Elbow Surg.

[2] Tamai K, Akutsu M, Yano Y (2014). Primary frozen shoulder: brief review of pathology and imaging abnormalities. J Orthop Sci.

[3] Millar NL, Meakins A, Struyf F (2022). Frozen shoulder. Nat Rev Dis Primers.

[4] Franz A, Klose M, Beitzel K (2019). [Conservative treatment of frozen shoulder]. Unfallchirurg.

[5] Harsulkar SG, Khatri SM, Rao K, Iyer C (2017). Effectiveness of Gong’s mobilization in cervical spondylosis: a prospective comparative study. Int J Community Med Public Health.

[6] Gopinath Y (2018). Effect of Gong’s mobilisation versus muscle energy technique on pain and functional ability of shoulder in Phase II adhesive capsulitis. J Clin Diagn Res.

[7] K. Sivasubramaniyan, S. Arul Pragassame, B. Srinivasan (2022). Effectiveness of Gong’s mobilization on pain and functional ability in patient with Periarthritis shoulder. YMER.

[8] Harsulkar SG, Rao K, Iyer C (2013). Effectiveness of Gong’s Mobilization on shoulder abduction in adhesive capsulitis: a case study. Appl Med Res.

[9] Shrestha M, Joshi D (2020). Effect of Gong’s mobilization on pain, range of motion and disability in frozen shoulder: a pilot study. Int J Physiother.

[10] Binder AI, Bulgen DY, Hazleman BL, Roberts S (1984). Frozen shoulder: a long-term prospective study. Ann Rheum Dis.

[11] Gong W, Park G, Kim C (2012). Effects of Gong’s mobilization in the side-lying position on shoulder abduction. J Phys Ther Sci.

[12] Gong W, Lee H, Lee Y (2011). Effects of Gong’s mobilization applied to shoulder joint on shoulder abduction. J-STAGE.

[13] Dilip JR, Babu VK, Sai Kumar N, Akalwadi A (2016). Effect of Gong’s mobilization versus Mulligan’s mobilization on shoulder pain and shoulder medial rotation mobility in frozen shoulder. Int J Physiother.

[14] Neviaser AS, Hannafin JA (2022). Adhesive capsulitis: a review of current treatment. Intern J Health Sci.

